# Cost-effectiveness analysis of Atezolizumab plus bevacizumab vs. Rivoceranib plus Camrelizumab as first-line treatment for unresectable hepatocellular carcinoma

**DOI:** 10.3389/fpubh.2026.1749414

**Published:** 2026-06-05

**Authors:** Mengmeng Liu, Tong Liu

**Affiliations:** Department of Pharmacy, Harbin Medical University Cancer Hospital, Harbin, China

**Keywords:** Atezolizumab, Bevacizumab, Camrelizumab, cost-effectiveness, HCC, Rivoceranib

## Abstract

**Background:**

This study evaluated the cost-effectiveness of Atezolizumab plus Bevacizumab versus Rivoceranib plus Camrelizumab as first-line treatment of unresectable hepatocellular carcinoma (HCC) from the perspective of healthcare payers in China.

**Methods:**

An economic evaluation using a 3-state partitioned survival model assessed the cost-effectiveness of Atezolizumab plus Bevacizumab versus Rivoceranib plus Camrelizumab. The Kaplan–Meier curves for overall survival (OS) and progression-free survival (PFS) from two clinical trials were digitally extracted, and the Weibull models were applied at the end of the trials to extrapolate long-term survival. The cost and health utility data were sourced from the published literature.

**Results:**

The anticipated cost for Atezolizumab plus Bevacizumab exceeded that for Rivoceranib plus Camrelizumab (109,555.94 USD vs. 15,264.92 USD). The estimated utility for Atezolizumab plus Bevacizumab was decreased compared to that of Rivoceranib plus Camrelizumab (1.47 QALYs vs. 1.59 QALYs). The ICER was −742,858.34 USD/QALY, suggesting the Rivoceranib plus Camrelizumab treatment was cost-effective compared to Atezolizumab plus Bevacizumab as the first-line treatment for advanced or metastatic HCC patients.

**Conclusion:**

Rivoceranib plus Camrelizumab was cost-effective compared to Atezolizumab plus Bevacizumab as a first-line treatment for advanced or metastatic HCC patients in China.

## 1 Introduction

Hepatocellular carcinoma (HCC), the most common type of primary liver cancer, is the third most common cause of cancer-related death worldwide and a leading cause of death in patients with chronic liver disease and cirrhosis, accounting for an estimated 830,200 deaths globally in 2020 (1). HCC is projected to surpass colorectal cancer and breast cancer to become the 3rd leading cause of cancer-related death in the US by 2040 (2). Dramatic increases in HCC incidence have been observed in the US over the past three decades, though recent data suggest incidence rates have plateaued or begun to decrease, particularly among younger and middle-aged adults, with a Surveillance, Epidemiology and End Results (SEER) analysis through 2015 demonstrating a 6.2% per year decrease in individuals aged 40–49 years and 10.3% per year decrease in individuals aged 50–59 years. Conversely, incidence has continued to rise steadily since the mid-2000s among older adults aged >60 years, in both men and women, and across all racial and ethnic groups, except among Asian/Pacific Islanders aged >70 years (3). HCC incidence and mortality rates have been highest in East Asia, South Asia, and parts of Africa, with more than half of the world’s HCC cases and deaths occurring in East Asia, and 45% of the world’s cases and 47% of deaths occurring in China alone in the year 2020 (4). Liver cancer is the fourth most prevalent and the second most lethal cancer in China (5). Approximately 410,000 patients were newly diagnosed with liver cancer in China in 2020 (6). HCC constitutes the majority of primary liver cancer, accounting for 75–85% of total cases (7). Therefore, it is of great significance to explore an appropriate treatment for HCC in China.

According to the latest clinical guidelines and research evidence, early diagnosis and personalized treatment strategies are crucial for improving the prognosis of HCC patients. Surgical resection, liver transplantation, and local ablation remain the mainstays of curative treatment for early-stage HCC. For intermediate to advanced stages, transarterial chemoembolization and systemic therapy, including targeted agents and immunotherapy, have demonstrated significant clinical benefits (8). The National Comprehensive Cancer Network (NCCN) guideline (HCC, version 2, 2025) recommends that the first-line treatment for advanced or metastatic HCC patients includes Atezolizumab+Bevacizumab [8]. In China Liver Cancer Guidelines for the Diagnosis and Treatment of Hepatocellular Carcinoma (2024 Edition), the first-line treatment includes Atezolizumab+Bevacizumab, Rivoceranib plus Camrelizumab, and so on (9).

In 2020, based on the clinically meaningful and statistically significant improvements in overall and progression-free survival (PFS) with atezolizumab plus bevacizumab over sorafenib in the IMbrave150 primary analysis, the combination was approved in more than 70 countries as first-line treatment for unresectable HCC (10). PD-L1 was an immune checkpoint protein expressed on tumor cells and tumor-infiltrating immune cells. PD-L1 could mediate suppression of anticancer immunity by binding to its receptors PD-1 and B7-1 (also known as CD80). Atezolizumab was a humanized engineered IgG1 monoclonal antibody targeting PD-L1 and thus had a mechanism of action distinct from anti-PD-1 antibodies. In addition to blocking the PD-L1 and PD-1 interaction, which could reinvigorate suppressed immune cells to eliminate cancer cells, Atezolizumab blocks PD-L1 and B7-1 binding, which might further enhance immune responses (11). Cancer cells differed fundamentally from normal cells as a result of having acquired hallmark capabilities that enable tumor growth and progression. Due to their high metabolic demands, growing solid tumors depended on vascularization for the provision of nutrients and oxygen and the disposal of metabolic waste products. Vascularization could be promoted by angiogenesis, and the first available anti-angiogenic therapy was Bevacizumab, a humanized monoclonal antibody that binds to all circulating, soluble VEGF-A isoforms. By binding to VEGF-A, Bevacizumab prevented the interaction of VEGF-A with VEGFR and thereby inhibited the activation of VEGF signaling pathways that promoted neovascularization (12). When Atezolizumab and Bevacizumab were combined, the anti–programmed death-ligand 1 (PD-L1)-related efficacy of Atezolizumab might be enhanced by Bevacizumab’s reversal of vascular endothelial growth factor (VEGF)-mediated immunosuppression and promotion of T-cell infiltration of tumors (13, 14). Rivoceranib was an orally administered, small-molecule, tyrosine kinase inhibitor (TKI) that selectively bound to and strongly inhibited VEGFR-2, reducing VEGF-mediated endothelial cell migration, proliferation, and tumor microvascular density. Rivoceranib had also been shown to augment T-cell-mediated anti-tumor cytotoxicity (15). Camrelizumab (Jiangsu Hengrui Pharmaceuticals Co, Ltd), a humanized, selective IgG4-*κ* monoclonal antibody against PD-1, exerted antitumor activity in a wide range of tumors (16). In a phase 1 study in pre-treated HCC and gastric or gastrooesophageal junction cancer, camrelizumab plus rivoceranib showed encouraging anti-tumor activity and acceptable tolerability (17). Thus, our study compared the efficacy of Atezolizumab plus Bevacizumab and Rivoceranib plus Camrelizumab through network meta-analysis. Network meta-analysis was a technique for comparing multiple treatments simultaneously in a single analysis by combining direct and indirect evidence within a network of randomized controlled trials. Network meta-analysis might assist in assessing the comparative effectiveness of different treatments regularly used in clinical practice, and therefore has become attractive among clinicians. A valid network meta-analysis would satisfy the assumption of transitivity, that there were no systematic differences between the available comparisons other than the treatments being compared (18).

Decision makers and patients required more information to make reasonable decisions regarding cancer treatment, and cost represented a crucial factor (19). In recent years, expenditures on cancer care have increased, with cancer becoming an important health concern worldwide, especially in countries such as China, which have limited healthcare resources. Thus, the evaluation of the pharmacoeconomic profile of treatment regimens was increasingly crucial (19). Cost-effectiveness analysis was a principal tool in health economic evaluation that allowed for the analysis of both the cost of a specific medical intervention and the benefits that it provided (20). We have done a lot of pharmacoeconomic research in the field of cancer before by using the cost-effectiveness analysis (21–25). Our results could offer the most cost-effective regimen in the treatment of cancer. When the government of China wants to add some drugs to the national medical insurance drug catalog in the future, it could give priority to the entry of some related drugs. In addition, other countries could also refer to our results to purchase some drugs, although the economic environment of each country might be different. Nowadays, there are two clinical trials reporting the efficacy and safety of Atezolizumab plus Bevacizumab versus sorafenib and Rivoceranib plus Camrelizumab versus sorafenib as a first-line treatment of advanced or metastatic HCC, respectively (26, 27). However, there are few reports of their economic evaluation in the treatment of HCC. This study would apply network meta-analysis and cost-effectiveness analysis to conduct an economic evaluation of Rivoceranib plus Camrelizumab versus Atezolizumab plus Bevacizumab in advanced or metastatic HCC patients.

## 2 Methods and materials

### 2.1 Target population and treatment strategies

Our study adhered to the provisions of the Consolidated Health Economic Evaluation Reporting Standards (CHEERS) (28). The source of efficacy and safety data of Rivoceranib plus Camrelizumab versus Atezolizumab plus Bevacizumab was from two global, multicenter, open-label, phase 2 trials (IMbrave150 and CARES-310) (26, 27). IMbrave150 was a global, open-label, phase 3 trial. Patients with unresectable HCC who had not previously received systemic treatment were randomly assigned in a 2:1 ratio to receive either Atezolizumab plus Bevacizumab or Sorafenib until unacceptable toxic effects occurred or there was a loss of clinical benefit (10, 26). Patients aged 18 years or older with locally advanced or metastatic and/or unresectable HCC, an Eastern Cooperative Oncology Group (ECOG) performance status score of 0 or 1, and Child-Pugh class A liver function who had not previously received systemic therapy for liver cancer were recruited from 111 cancer centers in 17 countries and regions. In the experimental group, Atezolizumab (1,200 mg) was given intravenously on the first day, and Bevacizumab (15 mg/kg) was given intravenously on the second day. The dose of Bevacizumab was calculated according to the average body weight of the Chinese population (66.2 kg) (19). In the control group, Sorafenib (400 mg) was taken twice a day. Every 3 weeks was a treatment cycle. CARES-310 was a randomized, open-label, international phase 3 trial done at 95 study sites across 13 countries and regions worldwide. Patients with unresectable or metastatic HCC who had not previously received any systemic treatment were randomly assigned (1,1) to receive either Camrelizumab 200 mg intravenously every 2 weeks plus Rivoceranib 250 mg orally once daily or Sorafenib 400 mg orally twice daily (27). Eligible patients were aged 18 years or older with histopathologically or cytologically confirmed HCC; had Barcelona Clinic Liver Cancer stage B or C disease, which was not amenable to or had progressed after surgical or locoregional therapy; and had not previously received any systemic therapy. Other key inclusion criteria were at least one measurable lesion per Response Evaluation Criteria in Solid Tumors version 1.1 (RECIST 1.1), Child-Pugh class A liver function, ECOG performance status of 0 or 1, a life expectancy of 12 weeks or more, and adequate organ function (27).

### 2.2 Disease modeling

**Figure 1:**
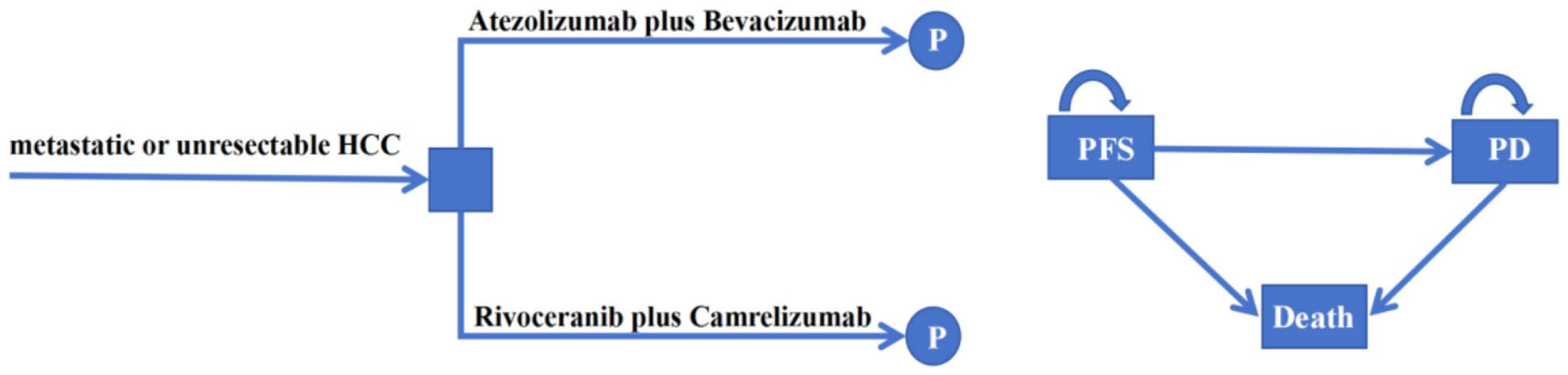
Model structure of a decision tree and a diagram of the partitioned survival model structure. **(A)** A decision tree; **(B)** Partitioned survival model structure with 3 health states. HCC, hepatocellular carcinoma; PFS, progression-free survival; PD, progressive disease.

**Figure 2:**
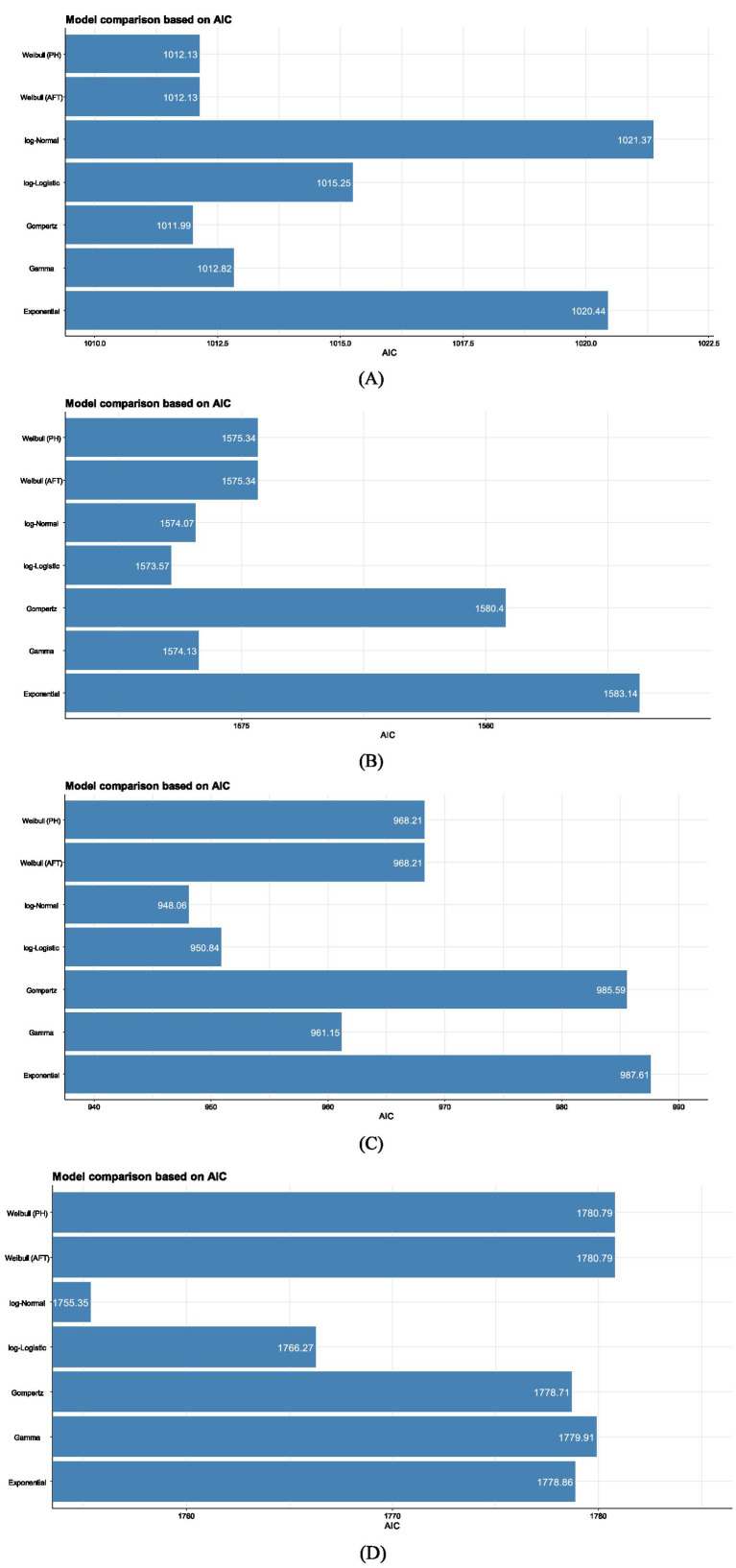
Akaike information criterion value in different models to select the most suitable survival model. **(A)** Seven models for OS in the Atezolizumab plus Bevacizumab arm; **(B)** seven models for OS in the Camrelizumab plus Rivoceranib arm; **(C)** seven models for PFS in the Atezolizumab plus Bevacizumab arm; **(D)** seven models for PFS in the Camrelizumab plus Rivoceranib arm. AIC, Akaike information criterion.

**Figure 3:**
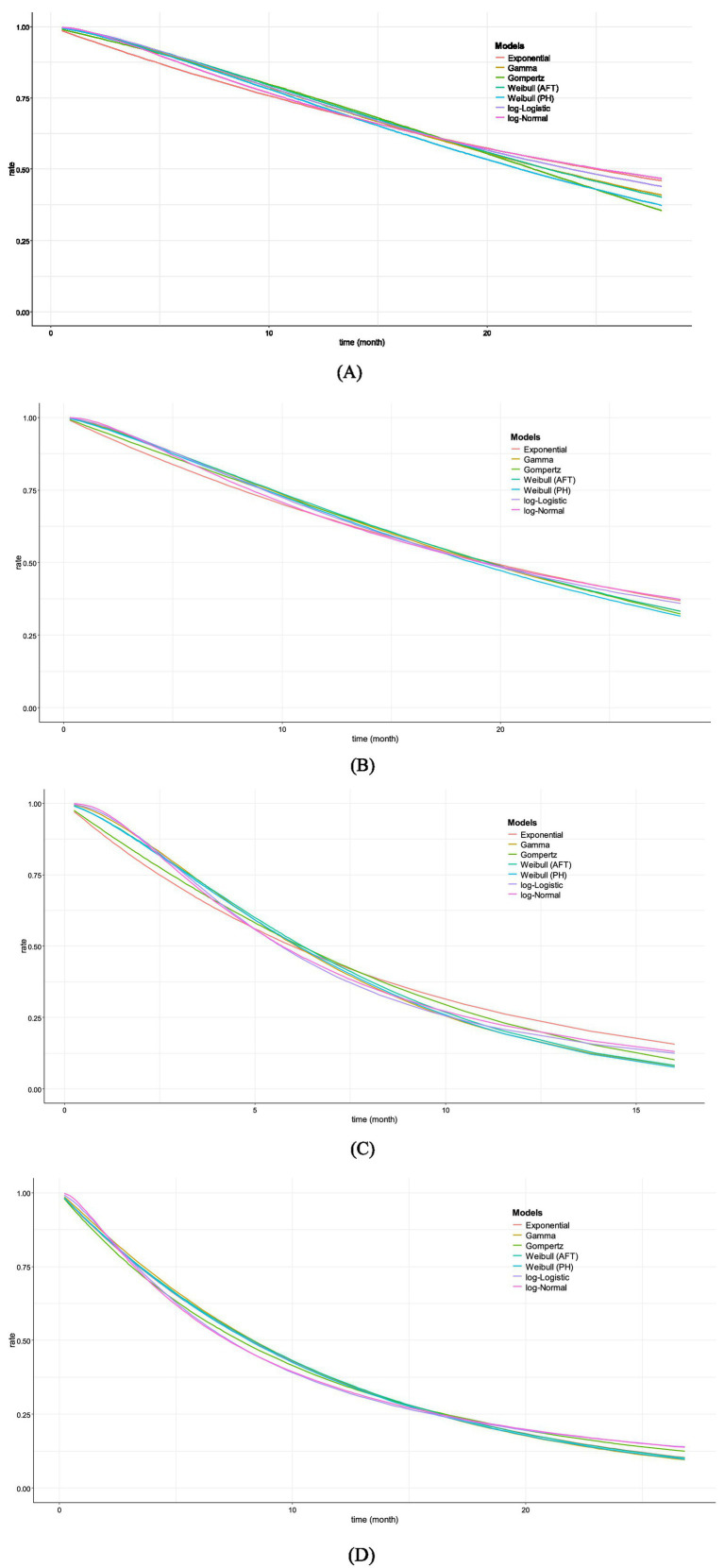
Model fitting diagrams. **(A)** OS curve in the atezolizumab plus bevacizumab arm; **(B)** OS curve in the camrelizumab plus rivoceranib arm; **(C)** PFS curve in the atezolizumab plus bevacizumab arm; **(D)** PFS curve in the camrelizumab plus rivoceranib arm.

**Figure 4:**
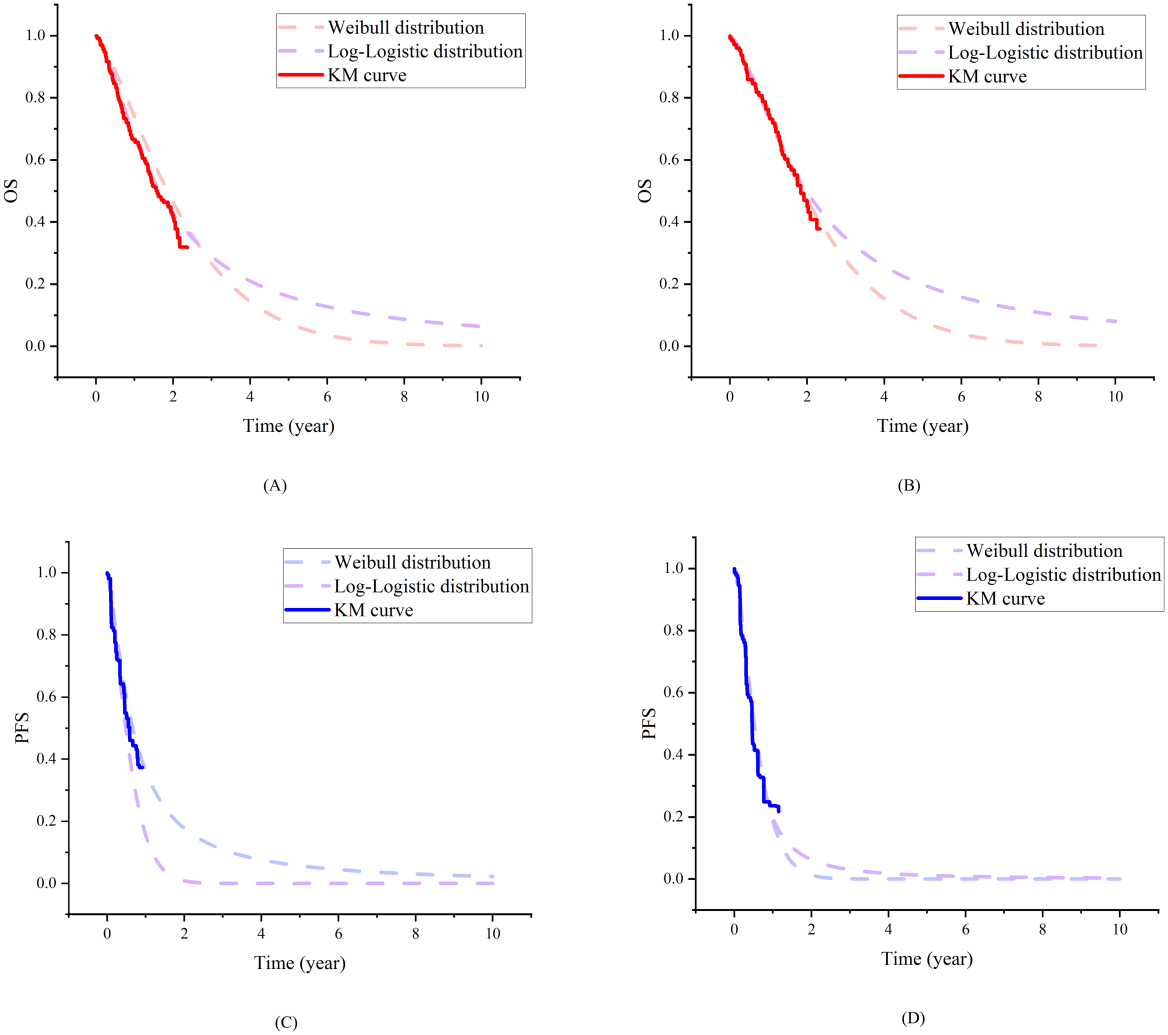
Kaplan–Meier, Weibull distribution, and log-logistic distribution curves to assess the plausibility of long-term extrapolations. **(A)** OS curve in the Atezolizumab plus Bevacizumab arm; **(B)** OS curve in the Camrelizumab plus Rivoceranib arm; **(C)** PFS curve in the Atezolizumab plus Bevacizumab arm; **(D)** PFS curve in the Camrelizumab plus Rivoceranib arm. KM, Kaplan–Meier; PFS, progression-free survival; OS, overall survival.

A partitioned survival model (PSM) was employed for this analysis. A PSM was a commonly used model in the economic evaluation of oncology drugs. The PSM used the area under the curve to represent the number of patients in each state. It was mainly used to evaluate the impact of interventions that could prolong the life of the patient on the expected lifetime and quality of life of the patient (29). Three health states were used by TreeAge Pro Healthcare 2022 software to frame the PSM in our study, including progression-free survival (PFS), progression disease (PD), and death, as illustrated in Figure 1. Software Getdata 2.25 was applied to extract and digitize the Kaplan–Meier curves. Seven distinct survival models were employed for the survival extrapolation from PFS and OS curves that have already been reported, such as Exponential, Gamma, Gompertz, Weibull (AFT), Weibull (PH), Log-Logistic, and Log-Normal models. Specifically, deducing the potential survival distribution from a Kaplan–Meier graph was advantageous for determining the parameters of these models (30). Selecting the most suitable survival model was based on the evaluation of all the fitted curves through a visualized approach, for example, the lowest Akaike information criterion value (AIC), the lowest Bayesian information criterion value (BIC), and software processing. The long-term clinical outcome survival function came from the fitting and extrapolation of the K-M curve. The goodness-of-fit was based on a visual inspection, AIC, and BIC (Figures 2, 3). Figure 3 shows the fitting curves of different models. The Weibull distribution was chosen due to its goodness-of-fit compared to the other models. When the Weibull distribution was applied, the curve showed that if the time horizon was 10 years, about 99.8% of the patients had passed away. Therefore, we chose a 10-year time horizon to simulate the entire life of the patients. To explore the uncertainty of the model, we also applied the Log-Logistic model to simulate the patients’ survival (Figure 4). The parameters of the Weibull curve and Log-Logistic curve are shown in Table 1.

**Table 1:** Distribution model of survival data.

Items	Weibull distribution	Log-logistic distribution
OS curve in the Atezolizumab plus Bevacizumab treatment	shape 1.24193; scale 0.01745	shape 1.4851; scale 19.0867
OS curve in the Rivoceranib plus Camrelizumab treatment	shape 1.33677; scale 0.01064	shape 1.509; scale 23.774
PFS curve in the Atezolizumab plus Bevacizumab treatment	shape 1.0137; scale 0.0817	shape 1.3957; scale 7.2584
PFS curve in the Rivoceranib plus Camrelizumab treatment	shape 1.3656; scale 0.0569	shape 1.883; scale 5.662

### 2.3 Costs and utility

From a payer perspective, this model exclusively included the direct expenditure for medical care services, such as the cost of drug fees (Atezolizumab, Bevacizumab, Rivoceranib, and Camrelizumab), the cost of follow-up care, the cost of disease management, and the management for serious adverse events (SAEs). The costs of Atezolizumab, Bevacizumab, Rivoceranib, and Camrelizumab were from the big data service platform for China’s health industry (31). Among these four drugs, only Atezolizumab was not included in the national basic medical insurance drug catalog of China, which indicated the costs of Bevacizumab, Rivoceranib, and Camrelizumab would be reimbursed by more than 75%. We also conducted a scenario analysis based on the situation of medical insurance. The costs of follow-up care, disease management, and SAEs management were derived from the published literature (32–36). In our study, adverse drug reactions with an incidence rate exceeding 5% and a grade surpassing or equal to grade 3 were selected to calculate the costs of SAEs management for the Atezolizumab plus Bevacizumab arm and Rivoceranib plus Camrelizumab arm. Among them, the costs of SAEs involved hypertension, aspartate aminotransferase (AST) increased, thrombocytopenia, hand-foot syndrome, and neutropenia (26, 27). The health utility index referred to an aggregate indicator of physical health with weights varying from different states (37). A range from 0 to 1 (0 denoting death and 1 denoting perfect health) was commonly used for the utility scale (19). Given the absence of quality-of-life statistics from the clinical trials, the utility values for the PFS and PD of advanced or metastatic HCC were sourced from the previously published literature, as the characteristics of the patients included in the literature were basically similar to those of the patients in this study (32). Based on the China Guidelines for Pharmacoeconomic Evaluations (2020 Version), discounting was recommended for studies with a time horizon longer than 1 year. Pharmacoeconomics evaluations should discount costs and health outcomes that occur in the future. It was recommended 5% per year to be used as the discount rate for the base case and 0–8% in the sensitivity analysis (38) (Tables 2, 3). The cost-effectiveness was assessed by calculating the incremental cost-effectiveness ratio (ICER), which measured the ratio between cost increments and quality-adjusted life years (QALYs) increments.

**Table 2:** Cost inputs in the PSM (USD).

Items	Date (Year)	Country	Cost per cycle	Low	High	Distribution	Reference
Atezolizumab	2025	China	4600.28	3680.22	5520.34	Gamma	(31)
Bevacizumab	2025	China	1386.72	1109.37	1664.06	Gamma	(31)
Rivoceranib	2025	China	411.09	328.87	493.30	Gamma	(31)
Camrelizumab	2025	China	361.38	289.10	433.66	Gamma	(31)
Follow-up care	2024	China	85.01	68.01	102.01	Gamma	(32)
Disease management	2024	China	853.00	682.40	1023.60	Gamma	(33)
Hypertension	2024	China	542.62	434.1	651.14	Gamma	(34)
AST increased	2023	China	765.11	612.09	918.13	Gamma	(35)
Thrombocytopenia	2022	China	0.16	0.13	0.19	Gamma	(36)
Hand-foot syndrome	2024	China	1789.55	1431.64	2147.46	Gamma	(34)
Neutropenia	2023	China	1185.92	948.74	1423.1	Gamma	(35)

**Table 3:** Other inputs in the PSM.

Items	Value	Low	High	Distribution	Reference
Utility (QALYs)					
PFS	0.76	0.68	0.84	Beta	(32)
PD	0.68	0.61	0.75	Beta	(32)
Other					
Discounting rate	0.05	0	0.08	Fixed	(38)
SAEs rate in Atezolizumab plus Bevacizumab arm					
Hypertension	0.12	0.10	0.14	Beta	(26)
AST increased	0.05	0.04	0.06	Beta	(26)
SAEs rate in Rivoceranib plus Camrelizumab arm					
Hypertension	0.38	0.30	0.46	Beta	(27)
AST increased	0.16	0.13	0.19	Beta	(27)
Thrombocytopenia	0.11	0.09	0.13	Beta	(27)
Hand-foot syndrome	0.12	0.10	0.14	Beta	(27)
Neutropenia	0.06	0.05	0.07	Beta	(27)

PFS, progression-free survival; PD, progressive disease; QALYs, quality adjusted life years; AST, aspartate aminotransferase; SAEs, serious adverse events.

### 2.4 Sensitivity analysis

In this study, both one-way sensitivity analysis and probabilistic sensitivity analysis (PSA) were employed for uncertainty measurement and robustness evaluation of the model. Among them, the costs and the incidence rates of SAEs in the one-way sensitivity analysis ranged within ±20% of their baseline values, while the utilities ranged within ±10% (Tables 2, 3). A Monte Carlo simulation was performed 1,000 times for PSA, with each iteration randomly sampling from the distributions of all parameters. To illustrate the uncertainty of potential willingness-to-pay (WTP) thresholds, the cost-effectiveness acceptability curves were employed (39). The WTP threshold in the cost-effectiveness acceptability curves was set at 40,000 USD/QALY, which was three times the per capita GDP of China in 2024 from the National Bureau of Statistics (38). In cost-effectiveness analysis, the WTP threshold value for QALY was recommended to be 1–3 times the GDP per capita (38). The WTP threshold was defined by the WHO as a value representing an estimate of what a consumer of healthcare might be prepared to pay for the health benefit. These thresholds were often based on cost-effectiveness ratios estimating health gains for the resources expended (40). The cost parameters were modeled using a gamma distribution, whereas the utility value parameters and SAE rate parameters were modeled with a beta distribution (41).

## 3 Results

### 3.1 Base case results

As depicted in Table 4, the anticipated cost for Atezolizumab plus Bevacizumab exceeded that for Rivoceranib plus Camrelizumab (109,555.94 USD vs. 15,264.92 USD). The estimated utility for Atezolizumab plus Bevacizumab was decreased compared to that of Rivoceranib plus Camrelizumab (1.47 QALYs vs. 1.59 QALYs). The ICER was −742,858.34 USD/QALY, suggesting the Rivoceranib plus Camrelizumab treatment was cost-effective compared to Atezolizumab plus Bevacizumab as the first-line treatment for advanced or metastatic HCC patients.

**Table 4:** Economic evaluation results.

Different Scenario	Cost (USD)	Utility (QALYs)	IC	IE	ICER (USD/QALYs)
			(USD)	(QALYs)	
Weibull distribution-10 years					
Atezolizumab plus Bevacizumab	109,555.94	1.47	94,291.02	−0.13	−742,858.34
Rivoceranib plus Camrelizumab	15,264.92	1.59	/	/	/
Weibull distribution-Using Basic medical reimbursement					
Atezolizumab plus Bevacizumab	93,202.79	1.47	82,545.66	−0.13	−650,324.21
Rivoceranib plus Camrelizumab	10,657.13	1.59	/	/	/
Log-Logistic distribution-10 years					
Atezolizumab plus Bevacizumab	115,887.21	1.90	97,650.52	−0.21	−461,492.62
Rivoceranib plus Camrelizumab	18,236.69	2.11	/	/	/
Weibull distribution-5 years					
Atezolizumab plus Bevacizumab	107,615.35	1.41	92,409.90	−0.11	−836,318.61
Rivoceranib plus Camrelizumab	15,205.45	1.52	/	/	/
SAEs did not occur during the treatment					
Atezolizumab plus Bevacizumab	109,550.19	1.47	94,471.24	−0.13	−744,278.23
Rivoceranib plus Camrelizumab	15,078.95	1.59	/	/	/
SAEs occurred in the first cycle					
Atezolizumab plus Bevacizumab	109,637.88	1.47	94,471.24	−0.13	−744,278.23
Rivoceranib plus Camrelizumab	15,078.95	1.59	/	/	/

USD, United States Dollar; QALYs, quality adjusted life years; IC, incremental cost; IE, incremental effectiveness; ICER, incremental cost-effectiveness ratio.

### 3.2 Sensitivity analyses

**Figure 5:**
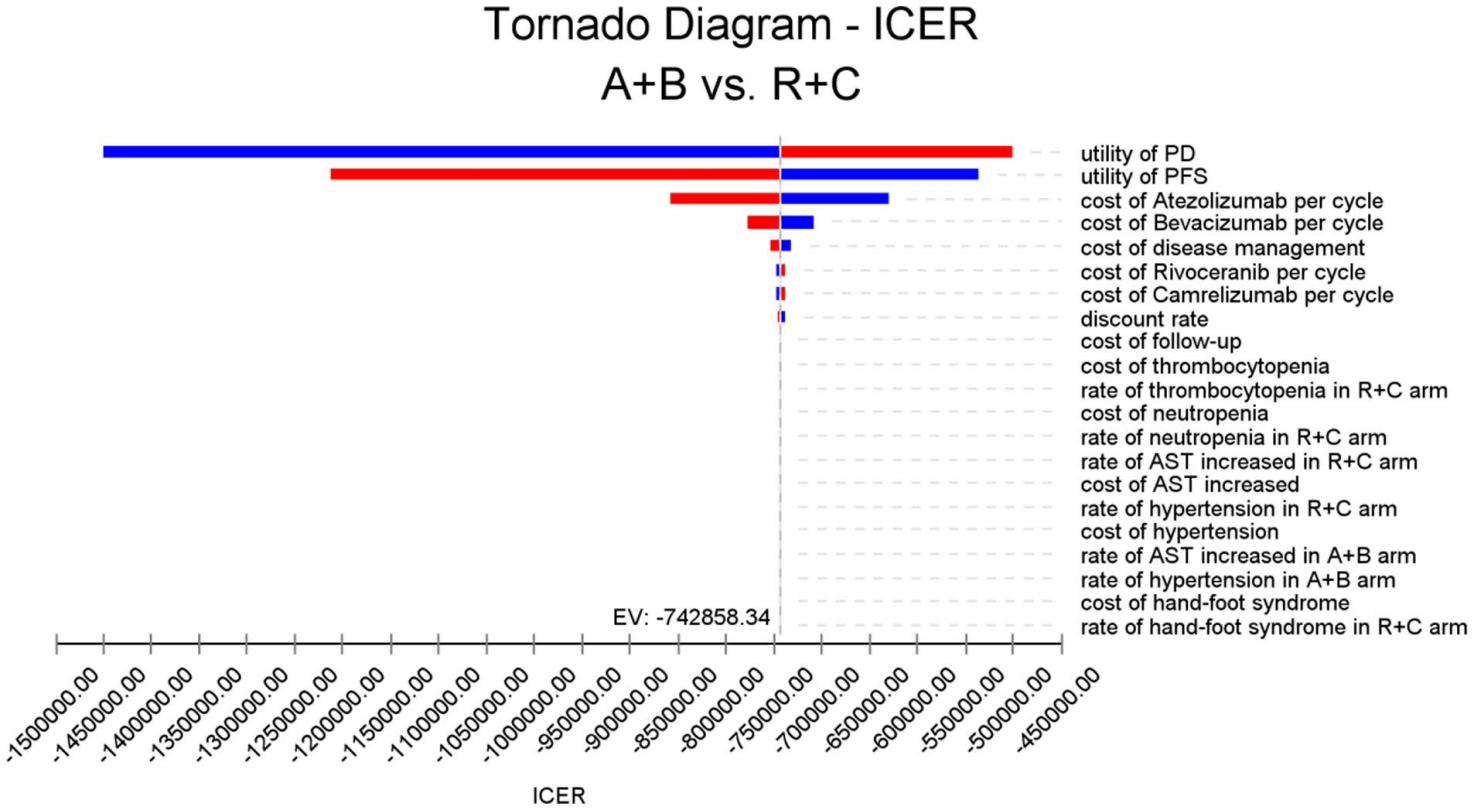
The tornado diagrams showed the outcome of the one-way sensitivity analysis. ICER, incremental cost-effectiveness ratio.

**Figure 6:**
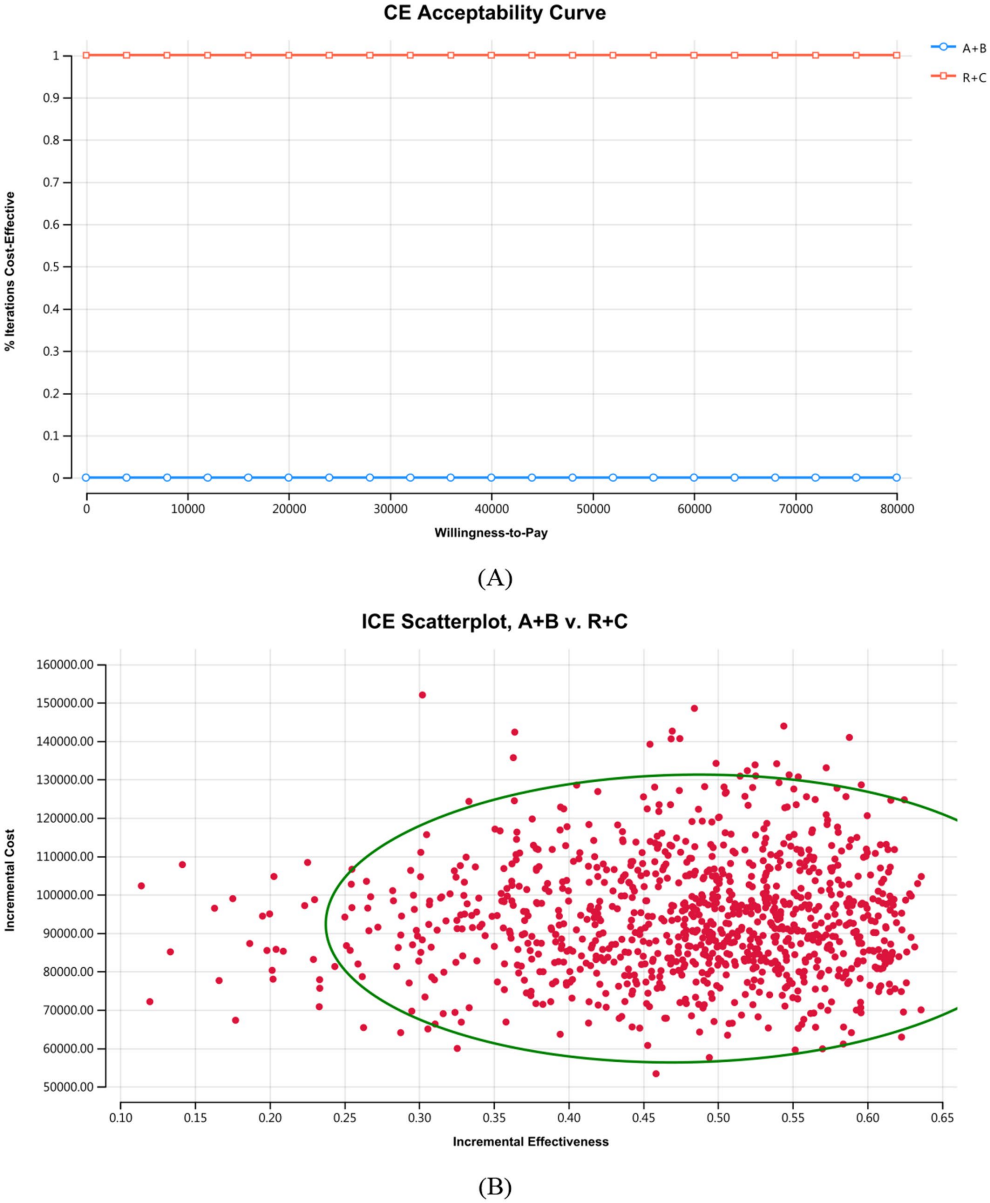
Probabilistic sensitivity analysis revealed that atezolizumab plus bevacizumab treatment was not cost-effective compared to rivoceranib plus camrelizumab treatment at the WTP threshold. **(A)** Cost-effectiveness acceptability curves: When the WTP threshold was 0–80,000 USD/QALY, atezolizumab plus bevacizumab treatment was not cost-effective; **(B)** Scatter plots: If there were 1,000 patients in the cohort at the beginning, none of the patients had benefit from the atezolizumab plus bevacizumab treatment.

The tornado diagram was employed to visualize the outcome of the one-way sensitivity analysis (Figure 5), revealing that the most influential factors on the ICER were the utilities of the PD and PFS, and the cost of Atezolizumab per cycle. Probabilistic sensitivity analysis revealed a 100% possibility that Atezolizumab plus Bevacizumab treatment was not cost-effective at the WTP threshold. The scatter plots indicated that at the threshold of 40,000 USD/QALY, there was a high possibility that the Atezolizumab plus Bevacizumab treatment was not cost-effective (Figure 6).

When the basic medical reimbursement of China was applied, the anticipated cost for Atezolizumab plus Bevacizumab exceeded that for Rivoceranib plus Camrelizumab (93,202.79 USD vs. 10,657.13 USD). The estimated utility for Atezolizumab plus Bevacizumab was decreased compared to that of Rivoceranib plus Camrelizumab (1.47 QALYs vs. 1.59 QALYs). The ICER was −650,324.21 USD/QALY, suggesting the Rivoceranib plus Camrelizumab treatment was cost-effective compared to Atezolizumab plus Bevacizumab as the first-line treatment for advanced or metastatic HCC patients.

When the Log-Logistic model was applied, the anticipated cost for Atezolizumab plus Bevacizumab exceeded that for Rivoceranib plus Camrelizumab (115,887.21 USD vs. 18,236.69 USD). The estimated utility for Atezolizumab plus Bevacizumab was decreased compared to that of Rivoceranib plus Camrelizumab (1.90 QALYs vs. 2.11 QALYs). The ICER was −461,492.62 USD/QALY, suggesting the Rivoceranib plus Camrelizumab treatment was cost-effective compared to Atezolizumab plus Bevacizumab as the first-line treatment for advanced or metastatic HCC patients.

When the time horizon of the model was 5 years, the anticipated cost for Atezolizumab plus Bevacizumab exceeded that for Rivoceranib plus Camrelizumab (107,615.35 USD vs. 15,205.45 USD). The estimated utility for Atezolizumab plus Bevacizumab was decreased compared to that of Rivoceranib plus Camrelizumab (1.41 QALYs vs. 1.52 QALYs). The ICER was −836,318.61 USD/QALY, suggesting the Rivoceranib plus Camrelizumab treatment was cost-effective compared to Atezolizumab plus Bevacizumab as the first-line treatment for advanced or metastatic HCC patients, which was consistent with that in a 10-year time horizon.

When all the SAEs occurred in both treatment arms, the anticipated cost for Atezolizumab plus Bevacizumab exceeded that for Rivoceranib plus Camrelizumab (109,550.19 USD vs. 15,078.95 USD). The estimated utility for Atezolizumab plus Bevacizumab was decreased compared to that of Rivoceranib plus Camrelizumab (1.47 QALYs vs. 1.59 QALYs). When none of the SAEs occurred in both treatment arms, the anticipated cost for Atezolizumab plus Bevacizumab exceeded that for Rivoceranib plus Camrelizumab (109,637.88 USD vs. 15,078.95 USD). The estimated utility for Atezolizumab plus Bevacizumab was decreased compared to that of Rivoceranib plus Camrelizumab (1.47 QALYs vs. 1.59 QALYs). The results suggested the Rivoceranib plus Camrelizumab treatment was cost-effective compared to Atezolizumab plus Bevacizumab as the first-line treatment for advanced or metastatic HCC patients.

## 4 Discussion

**Figure 7:**
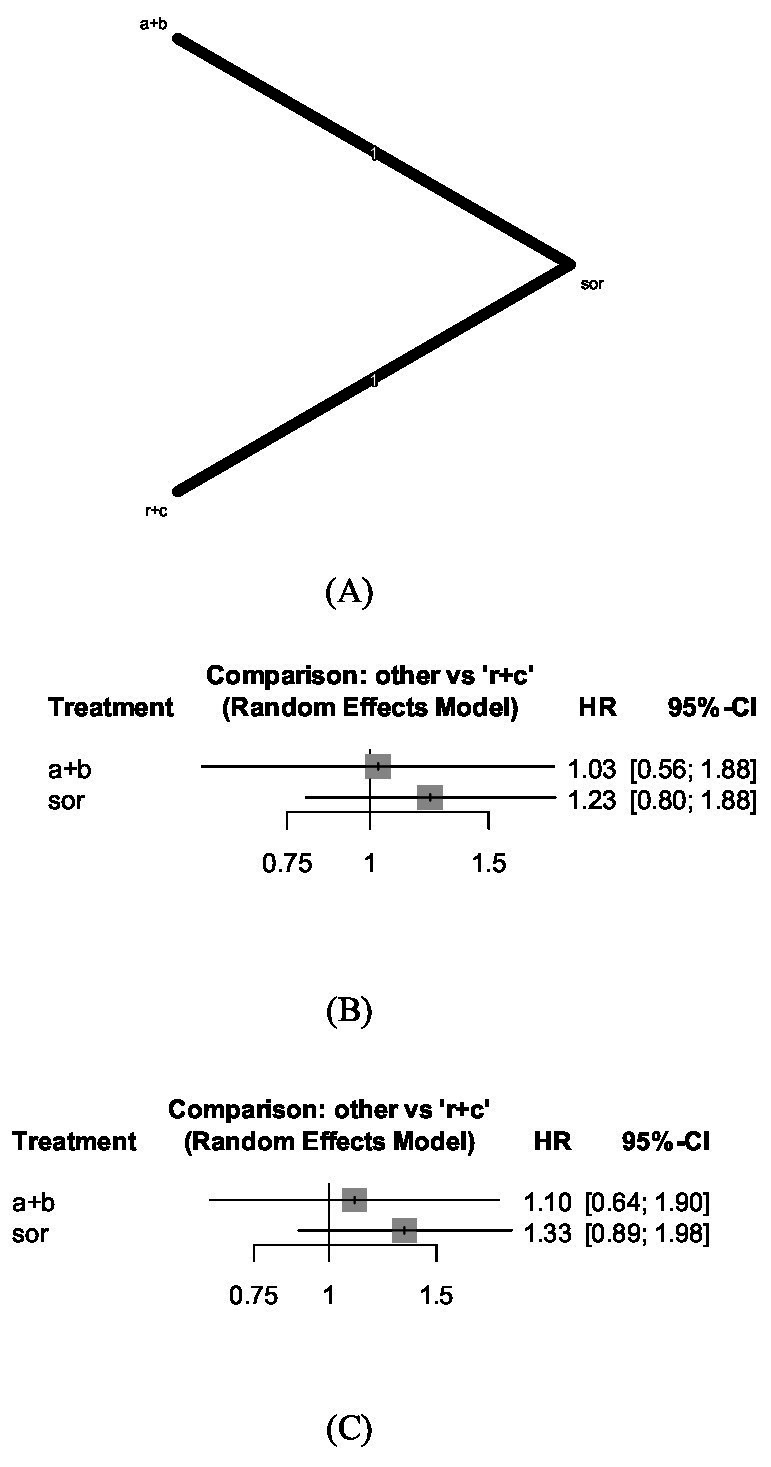
Network meta-analysis of the IMbrave150 trial and CARES-310 trial. **(A)** Network comparisons. **(B)** Forest plot of the network meta-analysis in OS. **(C)** Forest plot of the network meta-analysis in PFS. A + B, Atezolizumab plus Bevacizumab; R + C, Rivoceranib plus Camrelizumab; Sor, Sorafenib.

We firstly applied PSM to evaluate the cost-effectiveness of Atezolizumab plus Bevacizumab compared to Rivoceranib plus Camrelizumab as first-line treatment for advanced or metastatic HCC patients in China. The PSM divided the overall survival population into survival populations in different health states (such as PFS and PD). Therefore, PSM needed at least two survival curves to simulate the long-term survival of patients. The Markov model was also a commonly used method in pharmacoeconomic evaluation. It needed to consider the transition probability of patients in different health states at the same time, while PSM did not need to calculate the transition probability. PSM could directly obtain the distribution of the number of patients in each state from the survival curves, which was more convenient and simpler in calculation. In addition, when building a Markov model, some assumptions were usually made about the transition probability, which might be different from the actual observed data. For example, a constant natural mortality was sometimes used to replace the transition probability of switching to the death state, which might lead to the failure to correctly reflect the actual situation. The Markov model was derived from mathematical concepts to characterize stochastic processes. There was a classical “no memory” hypothesis, which stated that the future state was not affected by the past state. This feature made the model fail to consider the impact of the patient’s history, which could be avoided by using PSM. In general, the PSM was simpler and easier to calculate, and was closer to the actual observed data. PSM also had some limitations; for example, this method relied on the complete report of the survival curve in the clinical trial literature. In addition, PSM did not consider the specific transfer process between states. In general, the PSM was simpler and easier to calculate, and closer to the actual observed data. At present, it is increasingly applied to the pharmacoeconomic evaluation of advanced cancer treatment (42). Based on the NCCN guideline (HCC, version 2, 2025), the first-line therapy option was Atezolizumab plus Bevacizumab for advanced or metastatic HCC patients (8). While in Chinese guidelines, there were other regimens as first-line treatment for advanced or metastatic HCC patients, which included Rivoceranib plus Camrelizumab (9). Due to the lack of clinical trials directly comparing the efficacy and safety of Atezolizumab plus Bevacizumab versus Rivoceranib plus Camrelizumab, we collected the survival data of the Atezolizumab plus Bevacizumab arm and Rivoceranib plus Camrelizumab arm from two clinical trials (26, 27). Since the similar clinical characteristics and therapeutic schedules of the patients in the two clinical trials, we believed that two sets of data could be selected for indirect comparison. What’s more, to reduce the bias of indirect comparison, we conducted network meta-analysis to evaluate the hazard risk of Atezolizumab plus Bevacizumab versus Rivoceranib plus Camrelizumab (Figure 7). Through searching some databases, such as PubMed, we found there were only two studies reporting cost-effectiveness analysis of Atezolizumab plus Bevacizumab versus Rivoceranib plus Camrelizumab, which also applied network meta-analysis and Markov model to evaluate economic advantages. In our study, we used network meta-analysis to compare the efficacy of Atezolizumab plus Bevacizumab versus Rivoceranib plus Camrelizumab. Besides, we applied PSM to conduct cost-effectiveness analysis rather than the Markov model, as PSM was widely used in the pharmacoeconomic evaluation of patients with advanced metastatic cancer (43). Our results indicated that Rivoceranib plus Camrelizumab was cost-effective compared to Atezolizumab plus Bevacizumab as first-line treatment for metastatic HCC patients, which was similar to the previous studies, hoping to provide a reference for doctors, patients, and policymakers to decide on the treatment of advanced or metastatic HCC.

Based on our sensitive analysis, the results indicated that the most influential factors on the ICER were the utilities of the PD and PFS, and the cost of Atezolizumab plus Bevacizumab. As the cost of Rivoceranib plus Camrelizumab was lower, while the health utility of the patients using the Rivoceranib plus Camrelizumab was higher, the absolute advantage regimen was Rivoceranib plus Camrelizumab compared to Atezolizumab plus Bevacizumab. As the national basic medical insurance drug catalog of China included Bevacizumab, Rivoceranib, and Camrelizumab, indicating the costs of these three drugs could decrease by about 75%, we conducted a sensitivity analysis to explore the economic evaluation of the two treatment regimens when the national basic medical insurance was applied. The results showed Rivoceranib plus Camrelizumab treatment was cost-effective compared to Atezolizumab plus Bevacizumab. According to the survival data from the clinical trials, the simulation time in our study was 10 years, which could reflect about 99% of the patients’ lifetime in the Rivoceranib plus Camrelizumab treatment arm and Atezolizumab plus Bevacizumab treatment arm. To investigate the influence of simulation time, a sensitivity analysis was conducted with a simulation time of 5 years, which could reflect the lifetime of about 90% of patients in the Rivoceranib plus Camrelizumab treatment arm and Atezolizumab plus Bevacizumab treatment arm. Our results suggested that Rivoceranib plus Camrelizumab treatment was cost-effective compared to Atezolizumab plus Bevacizumab treatment as first-line treatment for advanced or metastatic HCC patients if the simulation time was 5 years (Table 4). As most adverse reactions of drugs occurred in the first cycle, and once SAEs occurred, patients needed to adjust the drug dosage or even terminate treatment, we assumed that all SAEs incurred during the first cycle, and the costs were calculated by multiplying the specific adverse reaction management price by the occurrence rate (44). To address the uncertainty of SAEs occurring, we conducted the scenario analysis. Our results showed Rivoceranib plus Camrelizumab treatment was cost-effective compared to Atezolizumab plus Bevacizumab treatment as first-line treatment for advanced or metastatic HCC patients, whether adverse reactions occur or not (Table 4).

Our study had several limitations. Firstly, this study was an indirect comparison from the cross-trial. As the patients’ survival data were not taken from a single, randomized controlled trial, there were some differences in baseline and clinical characteristics that could have impacted the results. The general limitations of indirect comparisons usually include potential bias resulting from different assessment times between studies and the comparability of endpoints used. To conduct the indirect comparison, we applied the network meta-analysis to simulate the long-term survival curves of different treatment arms. There might still be some bias from the inherent characteristics of the network meta-analysis, which could be corrected by the real-world data studies in the future. By comparing the baseline characteristics of patients in the two treatment arms, we found that there were two main differences between the two treatment arms. The patients in the Rivoceranib plus Camrelizumab arm were younger than those in the Atezolizumab plus Bevacizumab arm (58 vs. 64), indicating the prognosis of patients in the Atezolizumab plus Bevacizumab arm might be worse. The second difference was the geographical region of patients. The Rivoceranib plus Camrelizumab treatment was conducted mostly in Asia, while the Atezolizumab plus Bevacizumab treatment was conducted mainly in the rest of the world. Due to differences in race, there might be variations in treatment outcomes. Secondly, the utility values for health status were archived from previous literature, as the utility values of the advanced or metastatic HCC patients using Atezolizumab plus Bevacizumab or Rivoceranib plus Camrelizumab were not explicit. Our study obtained relevant quality-of-life scores of the patients through searching other literature, which could nearly reflect the health utility of the targeted patients. Thirdly, only the prices of SAEs were taken into consideration for this study, whose incidence rate surpassed 5%, and the adverse reaction grading level was equal to or exceeded 3. Although this approach was common in the cost-effectiveness analyses, lower-grade adverse events might also generate healthcare costs and affect quality of life, particularly in long-term immunotherapy treatments. In the sensitivity analysis, adverse reactions had a relatively minor impact on the robustness of the ICER; clinicians, patients, and policy-makers should consider the impact of low-grade adverse reactions when referring to the results of this study.

## 5 Conclusion

Rivoceranib plus Camrelizumab was cost-effective compared to Atezolizumab plus Bevacizumab as a first-line treatment for advanced or metastatic HCC patients in China.

## Data Availability

The datasets presented in this article are not readily available because NA. Requests to access the datasets should be directed to Tong Liu, 332581817@qq.com.

## References

[1] Sung H Ferlay J Siegel RL Laversanne M Soerjomataram I Jemal A . Global Cancer statistics 2020: GLOBOCAN estimates of incidence and mortality worldwide for 36 cancers in 185 countries. CA Cancer J Clin. (2021) 71:209–49. doi: 10.3322/caac.21660, 33538338

[2] Rahib L Wehner MR Matrisian LM Nead KT. Estimated projection of US Cancer incidence and death to 2040. JAMA Netw Open. (2021) 4:e214708. doi: 10.1001/jamanetworkopen.2021.4708, 33825840 PMC8027914

[3] Rich NE Yopp AC Singal AG Murphy CC. Hepatocellular carcinoma incidence is decreasing among younger adults in the United States. Clin Gastroenterol Hepatol. (2020) 18:242–248.e5. doi: 10.1016/j.cgh.2019.04.043, 31042582 PMC6817412

[4] Rich NE. Changing epidemiology of hepatocellular carcinoma within the United States and worldwide. Surg Oncol Clin N Am. (2024) 33:1–12. doi: 10.1016/j.soc.2023.06.004, 37945136 PMC12150546

[5] Zhou M Wang H Zeng X Yin P Zhu J Chen W . Mortality, morbidity, and risk factors in China and its provinces, 1990-2017: a systematic analysis for the global burden of disease study 2017. Lancet. (2019) 394:1145–58. doi: 10.1016/S0140-6736(19)30427-1 31248666 PMC6891889

[6] Rumgay H Arnold M Ferlay J Lesi O Cabasag CJ Vignat J . Global burden of primary liver cancer in 2020 and predictions to 2040. J Hepatol. (2022) 77:1598–606. doi: 10.1016/j.jhep.2022.08.021, 36208844 PMC9670241

[7] Xie D Shi J Zhou J Fan J Gao Q. Clinical practice guidelines and real-life practice in hepatocellular carcinoma: a Chinese perspective. Clin Mol Hepatol. (2023) 29:206–16. doi: 10.3350/cmh.2022.0402, 36545708 PMC10121293

[8] NCCN. Clinical Practice Guidelines in Hepatocellular Carcinoma. (2025). Version 2 [BD/OL]. Available online at: http://www.nccn.org (Accessed November 4, 2025).

[9] Zhou J Sun H Wang Z Cong W Zeng M Zhou W . China liver Cancer guidelines for the diagnosis and treatment of hepatocellular carcinoma (2024 edition). Liver Cancer. (2025) 14:1–57. doi: 10.1159/000546574, 41063733 PMC12503762

[10] Finn RS Qin S Ikeda M Galle PR Ducreux M Kim TY . Atezolizumab plus Bevacizumab in Unresectable Hepatocellular Carcinoma. N Engl J Med. (2020) 382:1894–905. doi: 10.1056/NEJMoa1915745, 32402160

[11] Rittmeyer A Barlesi F Waterkamp D Park K Ciardiello F von Pawel J . Atezolizumab versus docetaxel in patients with previously treated non-small-cell lung cancer (OAK): a phase 3, open-label, multicentre randomised controlled trial. Lancet. (2017) 389:255–65. doi: 10.1016/S0140-6736(16)32517-X 27979383 PMC6886121

[12] Garcia J Hurwitz HI Sandler AB Miles D Coleman RL Deurloo R . Bevacizumab (Avastin®) in cancer treatment: a review of 15 years of clinical experience and future outlook. Cancer Treat Rev. (2020) 86:102017. doi: 10.1016/j.ctrv.2020.102017, 32335505

[13] Hegde PS Wallin JJ Mancao C. Predictive markers of anti-VEGF and emerging role of angiogenesis inhibitors as immunotherapeutics. Semin Cancer Biol. (2018) 52:117–24. doi: 10.1016/j.semcancer.2017.12.002, 29229461

[14] Wallin JJ Bendell JC Funke R Sznol M Korski K Jones S . Atezolizumab in combination with bevacizumab enhances antigen-specific T-cell migration in metastatic renal cell carcinoma. Nat Commun. (2016) 7:12624. doi: 10.1038/ncomms12624, 27571927 PMC5013615

[15] Kang YK Ryu MH Di Bartolomeo M Chau I Yoon H Kim JG . Rivoceranib, a VEGFR-2 inhibitor, monotherapy in previously treated patients with advanced or metastatic gastric or gastroesophageal junction cancer (ANGEL study): an international, randomized, placebo-controlled, phase 3 trial. Gastric Cancer. (2024) 27:375–86. doi: 10.1007/s10120-023-01455-5, 38281295 PMC10896803

[16] Luo H Lu J Bai Y Mao T Wang J Fan Q . Effect of Camrelizumab vs placebo added to chemotherapy on survival and progression-free survival in patients with advanced or metastatic esophageal squamous cell carcinoma: the ESCORT-1st randomized clinical trial. JAMA. (2021) 326:916–25. doi: 10.1001/jama.2021.12836, 34519801 PMC8441593

[17] Xu J Zhang Y Jia R Yue C Chang L Liu R . Anti-PD-1 antibody SHR-1210 combined with Apatinib for advanced hepatocellular carcinoma, gastric, or Esophagogastric junction Cancer: an open-label, dose escalation and expansion study. Clin Cancer Res. (2019) 25:515–23. doi: 10.1158/1078-0432.CCR-18-2484, 30348638

[18] Rouse B Chaimani A Li T. Network meta-analysis: an introduction for clinicians. Intern Emerg Med. (2017) 12:103–11. doi: 10.1007/s11739-016-1583-7, 27913917 PMC5247317

[19] Liu T Yao R Tong Z Dong M Zhong L. Cost-effectiveness analysis of capecitabine versus active monitoring in stable or responding metastatic colorectal cancer after 16 weeks of first-line therapy. Ann Med. (2025) 57:2529573. doi: 10.1080/07853890.2025.2529573, 40616666 PMC12231269

[20] Lin YS O'Mahony JF van Rosmalen J. A simple cost-effectiveness model of screening: an open-source teaching and research tool coded in R. Pharmacoecon Open. (2023) 7:507–23. doi: 10.1007/s41669-023-00414-1, 37261616 PMC10333156

[21] Yao R Yao Y Teng X Jin Y Guan S Dong M . Cost-effectiveness analysis of adagrasib with or without cetuximab in the treatment of colorectal cancer patients with mutated KRAS G12C. Expert Rev Pharmacoecon Outcomes Res. (2025) 25:1453–61. doi: 10.1080/14737167.2025.2521439, 40518933

[22] Liu T Zhang X Liu B Yao Y Yao R Tong Z . Cost-effectiveness analysis of dostarlimab plus chemotherapy versus pembrolizumab plus chemotherapy as first-line treatment of metastatic non-squamous non-small cell lung cancer. J Chemother. (2025) 28:1–10. doi: 10.1080/1120009X.2025.2524906, 40580088

[23] Liu T Yao Y Liu B Teng X Dong M Zhang X. Cost-effectiveness analysis of FOLFOXIRI plus bevacizumab versus mFOLFOX6 plus bevacizumab as first-line treatment of metastatic colorectal cancer. Expert Rev Pharmacoecon Outcomes Res. (2025) 25:1427–35. doi: 10.1080/14737167.2025.2509706, 40395124

[24] Liu T Yao Y Teng X Tong Z Dong M Yao R. Cost-effectiveness analysis of sotorasib plus panitumumab in the treatment of refractory colorectal cancer with mutated KRAS G12C in the USA. Expert Rev Pharmacoecon Outcomes Res. (2025) 25:1463–71. doi: 10.1080/14737167.2025.2521444, 40518842

[25] Zhong L Dong M Liu T. Cost-effectiveness analysis of larotrectinib vs standard of care for treatment of metastatic NTRK fusion colorectal cancer. Expert Rev Pharmacoecon Outcomes Res. (2026) 26:253–66. doi: 10.1080/14737167.2025.2603943, 41384316

[26] Cheng AL Qin S Ikeda M Galle PR Ducreux M Kim TY . Updated efficacy and safety data from IMbrave150: Atezolizumab plus bevacizumab vs. sorafenib for unresectable hepatocellular carcinoma. J Hepatol. (2022) 76:862–73. doi: 10.1016/j.jhep.2021.11.030, 34902530

[27] Qin S Chan SL Gu S Bai Y Ren Z. Camrelizumab plus rivoceranib versus sorafenib as first-line therapy for unresectable hepatocellular carcinoma (CARES-310): a randomised, open-label, international phase 3 study. Lancet. (2023) 402:1133–46. doi: 10.1016/S0140-6736(23)00961-3, 37499670

[28] Husereau D Drummond M Augustovski F de Bekker-Grob E Briggs AH Carswell C . Consolidated health economic evaluation reporting standards 2022 (CHEERS 2022) statement: updated reporting guidance for health economic evaluations. Value Health. (2022) 25:3–9. doi: 10.1016/j.jval.2021.11.1351, 35031096

[29] Rui M Wang Y Fei Z Zhang X Shang Y Li H. Will the Markov model and partitioned survival model lead to different results? A review of recent economic evidence of cancer treatments. Expert Rev Pharmacoecon Outcomes Res. (2021) 21:373–80. doi: 10.1080/14737167.2021.1893167, 33691544

[30] Liu T Jin Y Dong M. Cost-effectiveness of Nivolumab plus Cabozantinib versus Cabozantinib as first-line treatment of metastatic renal cell carcinoma. Clin Genitourin Cancer. (2023) 21:e449–60. doi: 10.1016/j.clgc.2023.05.009, 37271697

[31] Yao ZH. The big data service platform for China’s health industry: Information Query of Drug Bid Winning. (2025). Available online at: https://data.yaozh.com/ (Accessed October 12, 2025).

[32] Liu L Wang L Ding Y Zhang Q Shu Y. Cost-effectiveness of atezolizumab plus bevacizumab versus sorafenib as first-line therapy in unresectable hepatocellular carcinoma in the US and Chinese setting: a modelling comparison study. BMJ Open. (2025) 15:e094804. doi: 10.1136/bmjopen-2024-094804, 40050065 PMC11887288

[33] Lian D Gan Y Xiao D Xuan D Liu S Wei Y. Cost-effectiveness of first-line systemic therapies for unresectable hepatocellular carcinoma. Br J Clin Pharmacol. (2024) 18:1–9. doi: 10.1111/bcp.16367, 39694066

[34] Huang Z Zhou L Zheng H Zhan M. Cost-effectiveness analysis of fruquintinib in Chinese patients with refractory metastatic colorectal cancer. Int J Clin Pharm. (2024) 46:872–80. doi: 10.1007/s11096-024-01721-1, 38642249

[35] Zhan M Huang Z Xu T Xu X Zheng H Wu F. Cost-effectiveness analysis of trastuzumab deruxtecan in patients with HER2-low advanced breast cancer based on DESTINY-Breast04. Front Public Health. (2023) 11:1049947. doi: 10.3389/fpubh.2023.1049947, 37457280 PMC10347396

[36] Jiang Y Wang X. Cost-effectiveness analysis of pembrolizumab plus standard chemotherapy versus chemotherapy alone for first-line treatment of metastatic non-squamous non-small-cell lung cancer in China. Eur J Hosp Pharm. (2022) 29:139–44. doi: 10.1136/ejhpharm-2020-002208, 32737070 PMC9047884

[37] Drummond MF O'Brien B Stoddart GL. Methods for the Economic Evaluation of Health Care Programmes. 2nd ed. Oxford, UK: Oxford University Press (1997).

[38] Liu GE Hu SL Wu JH. China Guidelines for Pharmacoeconomic Evaluations (2020 Version). Beijing: China Market Press (2020).

[39] Aguiar-Ibáñez R Hardern C van Hees F Lee D Patel A Chhabra N . Cost-effectiveness of pembrolizumab for the first-line treatment of patients with unresectable or metastatic MSI-H/dMMR colorectal cancer in the United States. J Med Econ. (2022) 25:469–80. doi: 10.1080/13696998.2022.2043634, 35184650

[40] Pron G Hwang M Nasralla M Smith R Cheung A Murphy K. Cost-effectiveness and willing-to-pay thresholds for vertebral augmentation of osteoporotic vertebral fractures, what are they based on: a systematic review. BMJ Open. (2023) 13:e062832. doi: 10.1136/bmjopen-2022-062832, 37491092 PMC10373718

[41] Courtney PT Yip AT Cherry DR Salans MA Kumar A Murphy JD. Cost-effectiveness of Nivolumab-Ipilimumab combination therapy for the treatment of advanced non-small cell lung cancer. JAMA Netw Open. (2021) 4:e218787. doi: 10.1001/jamanetworkopen.2021.8787, 33938936 PMC8094011

[42] Shao RJ Tang WX Ma AX. Application of partition survival model in pharmacoeconomic evaluation. China Health Econ. (2019) 38:60–3.

[43] Liu T Liu S Guan S Tai Y Jin Y Dong M. Cost-effectiveness analysis of pembrolizumab versus chemotherapy for microsatellite instability-high or mismatch repair-deficient metastatic colorectal cancer. J Chemother. (2023) 35:745–52. doi: 10.1080/1120009X.2022.2162220, 36591729

[44] Su D Wu B Shi L. Cost-effectiveness of Atezolizumab plus bevacizumab vs Sorafenib as first-line treatment of Unresectable hepatocellular carcinoma. JAMA Netw Open. (2021) 4:e210037. doi: 10.1001/jamanetworkopen.2021.0037, 33625508 PMC7905498

